# Preparation of chitosan nanoparticle containing recombinant StxB antigen of EHEC and evaluation its immunogenicity in BALB/c mice

**Published:** 2018-12

**Authors:** Pegah Almasian, Jafar Amani, Fahimeh Baghban Arani, Shahram Nazarian, Rouhollah Kazemi, Niloufar Mirzaee Tabrizi

**Affiliations:** 1 Department of Genetics and Biotechnology, School of Biological Sciences, Varamin-Pishva Branch, Islamic Azad University, Varamin, Iran; 2 Applied Microbiology Research Center, Systems Biology and Poisonings Institute, Baqiyatallah University of Medical Sciences, Tehran, Iran; 3 Department of Biology, Faculty of Sciences, Imam Hossein University, Tehran, Iran; 4 Department of Agricultural Biotechnology, National Institute of Genetic Engineering and Biotechnology (NIGEB), Tehran, Iran

**Keywords:** Enterohemorrhagic, *Escherichia coli*, Stx2B subunit, Nanoparticle chitosan, Subunit vaccine

## Abstract

**Background and Objectives:**

*Escherichia coli* O157:H7 is one of the most important food pathogens that produces colitis and bloody urine in humans. The Stx2B subunit is considered as one of the candidates for vaccine due to its immunogenic and adjuvant properties. Designing a mucosal vaccine using nanoparticles for protecting the antigen against degradation and controlling the release of antigen are important. The objective of the current study was to prepare nanoparticles containing the Stx2B subunit of *E. coli* O157:H7 and evaluation of its immunogenicity in the mouse model.

**Materials and Methods:**

*E. coli* BL21 DE3 and pET28a-stxB were used for expression of the *stx2b* gene. After inducing gene expression, purification of the Stx2b protein was performed. Then, chitosan nanoparticle containing recombinant Stx2B was prepared and administered to BALB/c mice. IgA and IgG titers in serum and IgA titers in feces of immunized and control mice were evaluated by the ELISA method.

**Results:**

After expression and purification of the Stx2B recombinant protein, an expected band of 13 kDa was observed on the SDS-PAGE gel and confirmed by Western Blot analysis. The size of the nanoparticle containing Stx2B was 290 nm. In the immunized mice, IgG and IgA titers were significantly increased. The immunized mice were challenged against *E. coli* O157:H7 Stx+ and the shedding analysis showed that colonization of bacteria in the intestinal tract decreased.

**Conclusion:**

Oral administration of nanoparticles containing Stx2B as a candidate for the vaccine can induce a systemic and mucosal immune response against Stx2 toxin and can provide acceptable protection.

## INTRODUCTION

The Gram-negative bacteria of *Escherichia coli* belong to the Enterobacteriaceae family as one of the most common pathogens of urinary, respiratory and gastrointestinal tracts infection. Moreover, nosocomial infections and diarrhea are highly associated with the emergence and prevalence of new strains (1, 2). The presence of this bacterium in the natural flora of the human intestine and hospital infections has suggested the emergence of antibiotic-resistant strains (3, 4). The bacterium has the ability to produce a toxic form, similar to Shiga toxin, and so far two types of these toxins have been identified as verotoxin 1 and 2. These toxins can make the bacteria pathogenic although all bacterial serotypes do not have the ability to produce both types of poisons (5, 6). Enterohemorrhagic *E. coli* (EHEC) has a complex antigenic system and is therefore classified based on its antigenic properties. The pathogenicity of this bacterium can be obtained through plasmids or other genetic elements that can be transmitted, and these capabilities enable the bacteria to adhere to specific cell types in the specific hosts or produce toxins (7). Like all other AB5 families of toxins, Stx consists of one subunit with enzyme activity (32 kDa) called StxA and five subunits of 7.7 KDa StxB, which together create a pentameric structure and are responsible for attaching the toxins to their receptors. The two subtypes A and B are placed side by side with noncovalent bonds, so that the C-terminal end of the subunit A is placed inside the pore formed by pentamer B. StxA has a highly specific N-glycosidase activity. The gene that encoded Shiga toxin is placed on similar lambda bacteriophages, which are highly mobile genetic elements. These elements play an important role in the horizontal transformation of genes and genome variation. This gene is located at the last locus in phage genome and downstream of the last promoter. When the lytic cycle is activated, Stx is expressed at high levels. Phage regulates the production of toxins through the activation of phage promoters, the proliferation of the copy numbers of the gene, and the secretion of the toxin (8). The specific receptor of Stx is Globotriaosylceramide (Gb3), a neutral sphingolipid, which is expressed at a high level in some eukaryotic cells, such as endothelial cells of the kidney and vein. The position of this receptor on the membrane surface is such that the trisaccharide region is attached to the StxB and the ceramide region is placed as a noncovalent bond among of membrane fatty acids (9). After the toxin recognized target cells that have high levels of the specific Gb3 receptor, the toxin binds to the receptor via the subunit B. Although AB5 toxins have used the same pathways to enter the cell to reach the target protein, Stx has some discrepancies in details. Unlike other toxins, Stx lacks a signal sequence to transfer to the endoplasmic reticulum (10). By activating some signal pathways, Stx toxin leads to cell disruption and apoptosis. The disruption of kidney endothelial cells cause kidney failure and in some case death. On the other hand, Stx, through the disruption of the endothelial layer of the blood vessels of the digestive tract, causes hemorrhagic colitis and bloodly urine which are symptoms of HUS. As a result, an inflammatory response occurs which in turn leads to increased expression of Stx and subsequent severity of the disease (8). The nanospheres such as chitosan have a matrix structure in which active substances, such as peptides, can be adsorbed onto their surface, or enclosed or dissolved in the matrix. Chitosan and its derivatives have mucosal adhesion property, so they can be used to increase the absorption of drugs and protein orally. It has been demonstrated that loading of drugs with micro and nanoparticles using chitosan enhance absorption at mucosal surfaces. Chitosan and its derivatives open the cellular connections and facilitate the intercellular transfer of drugs and proteins (11, 12). In the current study, we aimed to design and produce a nano-based vaccine candidate against *E. coli* O157: H7 and evaluate its immunogenicity in an animal model.

## MATERIALS AND METHODS

### Plasmids, bacterial strains and media.

pET-28a (+) plasmid (Novagen, USA), BL21 (DE3) (Pasteur Institute of Iran) were used in this study. Bacteria were grown in Luria-Bertani (LB) broth or on LB agar supplemented with kanamycin (SIGMA, 100 g/ml) when needed.

### Confirmation of pET-28a plasmid harboring *stx2b* gene.

The PCR reaction was performed using the Taq DNA polymerase and specific primer. The following program was used for amplification of *stx2b* fragment: Initial denaturation at 94°C for 5 min, 30 cycles at 94°C 30s, 64°C for 30s, 72°C for 1 min and final extension at 72°C for 10 min. PCR product was observed on 1% agarose gel.

### Expression and purification of the recombinant Stx2B protein.

In order to evaluate *stx2b* expression, five recombinant colonies of the BL21DE3, carrying the recombinant plasmid pET28a, were grown at 37°C (LB broth containing 50 μg/mL kanamycin) to an OD_600_ of 0.6. Then, IPTG was added to the culture medium at a final concentration of 1 mM and incubated for 5 hours at 37°C and 150 rpm in a shaker. The medium was centrifuged at 5,000 rpm for 5 minutes and 4 ml of lysis buffer (100 mM NaH_2_PO_4_, 10 mM Tris-Cl, 8 M urea, pH 8.0) was added to the sediment and mix thoroughly. After sonication (6 times for 10 second with high power), the lysate was centrifuged (15 min, 10000 g, 4°C) and the supernatant was applied on a Ni-NTA affinity chromatography column (Qiagen).

The recombinant protein appeared in inclusion bodies after analysis on 12% SDS-PAGE, and denaturing condition was used for purification. The purification steps were performed according to the manufacturer instructions. Nickel-nitrilotriacetic acid (Ni-NTA) column was equilibrated with lysis buffer and the protein solution was loaded onto the column at a flow rate of 0.5 ml/min. The impurity was removed two times by washing the column with washing buffer (100 mM NaH_2_PO_4_, 10 mM Tris-Cl, 8 M urea pH=5.9). The protein was eluted with elution buffer (100 mM NaH_2_PO_4_, 10 mM Tris-Cl, 8 M urea) at pH=4.5. Protein concentration was determined by the Bradford method with BSA (Bovine Serum Albumin) as a standard.

### Western blotting to confirm expressed protein.

The recombinant protein was separated by 12% SDS-PAGE and transferred onto PVDF membrane (Roche). The membrane was blocked with 5% skimmed milk in TBS buffer (50 mM Tris-Cl, 150 mM NaCl, pH=7.5) containing 0.05% Tween 20 (37°C, 2 hours). The membrane was incubated with HRP-conjugated mouse poly His-tag antibody (1:2000 Roche). Finally, the membrane was soaked in 3, 3′-Diaminobenzidine tablet (DAB Reagents; Sigma) for signal development.

### Preparation of chitosan nanoparticles.

50 mg chitosan was dissolved in a final volume of 25 ml of acetic acid 2% and placed on a stirrer for 1 hour at room temperature to completely dissolve; then filtered by 0.45-micron filter. The purified protein (rStx2B) was added drop wise to 7.5 ml of chitosan solution for 10 minutes and placed on the stirrer for 3 minutes until it was thoroughly mixed. Then pH adjusted on 5.5 by NaOH (1M) remains on the stirrer for 30 minutes. In the next step, 5 ml of TPP was added gradually to the solution containing chitosan and purified protein. Sonication was performed (3 times for 20 second with high power) to prevent the accumulation of nanoparticles. A solution was prepared as negative control under the same conditions. Finally, the solutions were centrifuged at 13000 rpm for 45 minutes at 4°C.

### Determination of loading percentage.

100 μl of supernatant was separated and the concentration of recombinant protein measured. The percentage of loaded protein on the nanoparticle was calculated based on the following formula. Moreover, Bradford test was used for determination the release rate of the rStx2B from nanoparticles.

### Determination of nanoparticles size.

The size of nanoparticles was measured by a particle size analyzer. This device, using dynamic light distribution (DLS), measures size according to the Brownian motion of nanoparticles.

### Animal model immunization.

To determine the antigenicity of the recombinant Stx2B, fifteen BAL-B/c mice (female, 6–7 weeks old, Pasteur Institute, Tehran, Iran) were randomly divided into four groups A to D) and acclimatized for 1 week. Group A was respectively inoculated with 100 μg of nanoparticle Stx2B subcutaneously into the quadriceps muscle of mice. The group B and C were immunized via oral and oral-injection route with 100 μg of nanoparticle Stx2B, respectively. Group D was immunized with PBS as the negative control. Immune serum was prepared from the blood sample of each mice group blood was transferred to vials and allowed to clot for 30 min, then serum was collected by centrifugation) and frozen at −70°C until use. The serum samples of each mice group were prepared and then pooled for immunological analyses.

### Determination of Igg and IgA by ELISA.

The collected sera were subjected to ELISA-based antibody titer assays. The purified rStx2B (500 ng/well) were used to load Maxisorb plates (Nunc, Denmark) with 100 μl bicarbonate buffer (15 mM Na_2_CO_3_ and 35 mM NaHCO_3_) incubated 2 h at 37°C. The wells were blocked for 2 hours at 37°C by the addition of 200 μl of 5% (w/v) skimmed milk in PBST (PBS containing 0.05% Tween-20) and washed three times with PBST. The wells incubated with serially diluted serum from immunized mice in triplicate at 100 μl/well for 1 hour at 37°C. The bound antibodies were detected with HRP-conjugated goat anti-mouse IgG (Sigma) and HRP-conjugated rabbit anti-mouse IgA (Sigma) in a 1:5000 dilution for 1 hour and washed three times with PBST (13). The reaction was developed with O-phenylenediamine (OPD) as a substrate (Sigma) for 15 min at room temperature in the dark. H_2_SO_4_ (2.5 M) was used to stop the reaction and the absorbance score was measured at 492 nm in an ELISA reader.

### Challenges investigation in immunized mice.

In order to determine that the Stx2B-specific antibodies in immunized mice serum could reduce or prevent the *E. coli* O157:H7 shedding in feces, subcutaneously immunized and non-immunized control mice were infected orally with 10^9^ CFU of *E. coli* O157:H7. The fecal samples of control and test mice were collected for two weeks. Thus, 100 mg of fecal samples were weighted and cultured in f liquid LB at 37°C for 2 h. Serial dilutions of supernatant were prepared and cultured on Sorbitol McCanky Agar medium. Plates were kept at 37°C for 15 to 18 hours, and then white colonies were counted.

## RESULTS

### Confirmation of pET-28a plasmid containing *stx2b* gene using PCR technique.

**Fig. 1.**
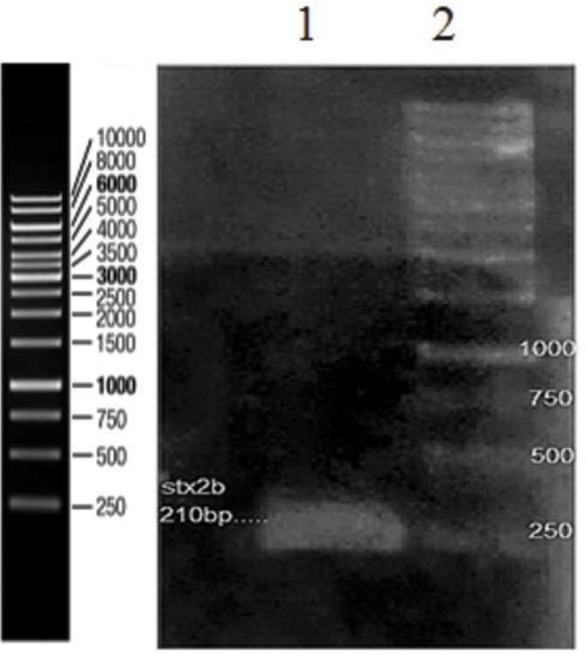
The presence of stx2b gene in the pET28a plasmid on agarose gel 1%. line 1: Control Sample, lane 2: Stx2b gene (210 bp), lane 3: DNA size marker

To ensure for the presence of *stx2b* gene in the pET28a plasmid, PCR was performed with T7 universal primers. The *stx2b* fragment (210 bp) was observed on 1% agarose gel (Fig. 1).

### Expression and Purification of the Stx2B recombinant protein.

**Fig. 2.**
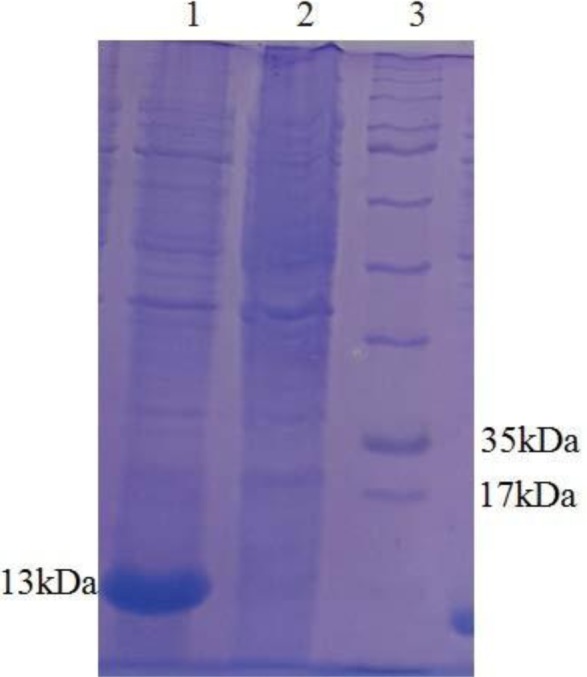
SDS-PAGE analysis of the recombinant Stx2b protein on SDS-PAGE gel 12%. lanes 1; pET28a-Stx2B (13 kDa), lanes 2; before induction, lanes 3; Protein size Marker.

**Fig. 3.**
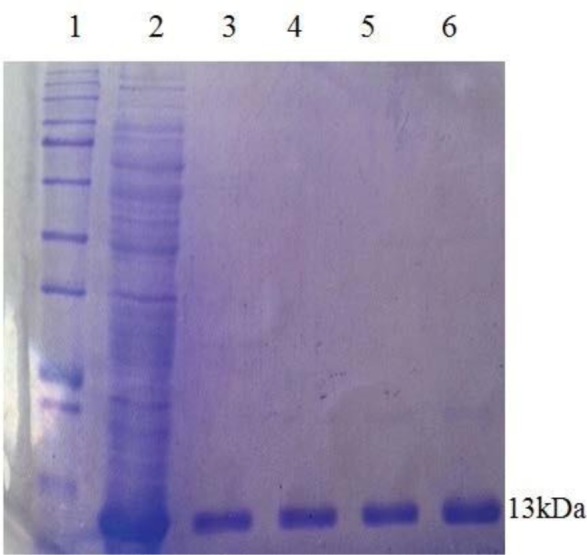
Purification of the recombinant Stx2B protein using the Ni-NTA column. lanes 1; Protein size markers, lanes 2; Flow lanes 3; wash, lanes 4; Elution 1 (pH = 4.5), lines 5; Elution 2 (pH = 4.5), lines 6; Elution 3 (pH = 4.5).

Expression of recombinant protein was analyzed on 12% SDS-PAGE and desired rStx2B (13 kDa) in fusion with 6x His-tag (N-terminal) were detected (Fig. 2). The recombinant protein was produced as inclusion bodies (IB) which subsequently solubilized using 8 M urea and purified using Ni-NTA column (Fig. 3).

### Western blotting to confirm protein expression.

**Fig. 4.**
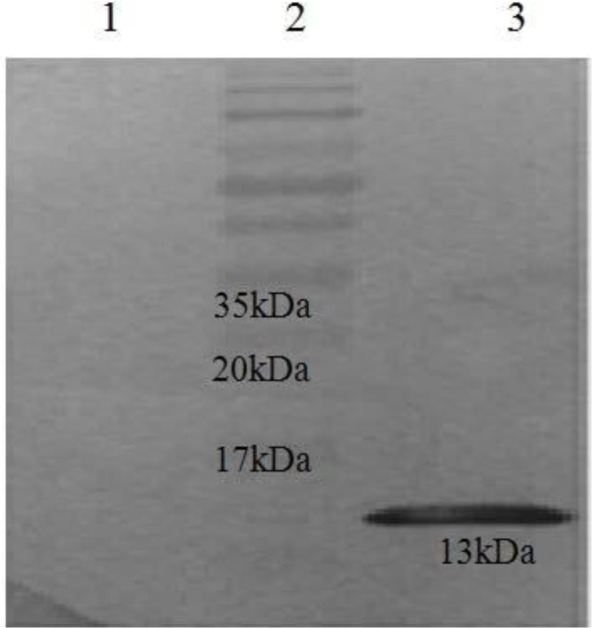
Confirmation of Stx2B protein using western blotting. lanes 1; Sample without Stx2B as control, lanes 2; Protein size marker, lanes 3; recombinant Stx2B protein

The authenticity of recombinant Stx2B (13 kDa) proteins were confirmed by poly His-tag antibody. In contrast, no reactivity was observed in negative control (Fig. 4).

### Determining the size of the nanoparticle using Zeta sizer.

**Fig. 5.**
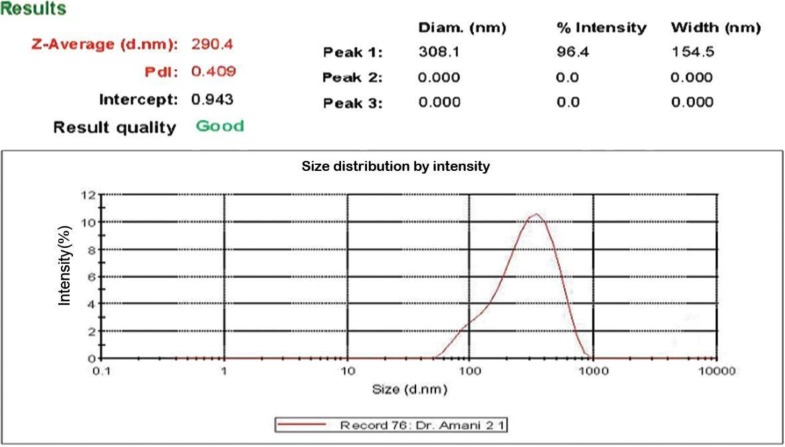
Results of DLS data for chitosan nanoparticle size at a suspended buffer. Based on the curve nanoparticles can be seen in a one size.

The Zeta particle potential is an important factor in the stability of the particles in solution. Chitosan and three methyl chitosan particles with zeta-positive potential have greater stability in solution. Moreover, higher zeta potential of chitosan lead to the adhesion particles to proteins and the mucosal surface and has a better ability to deliver protein in mucosal immune system (Fig. 5).

### Evaluation of immunogenicity using ELISA.

**Fig. 6.**
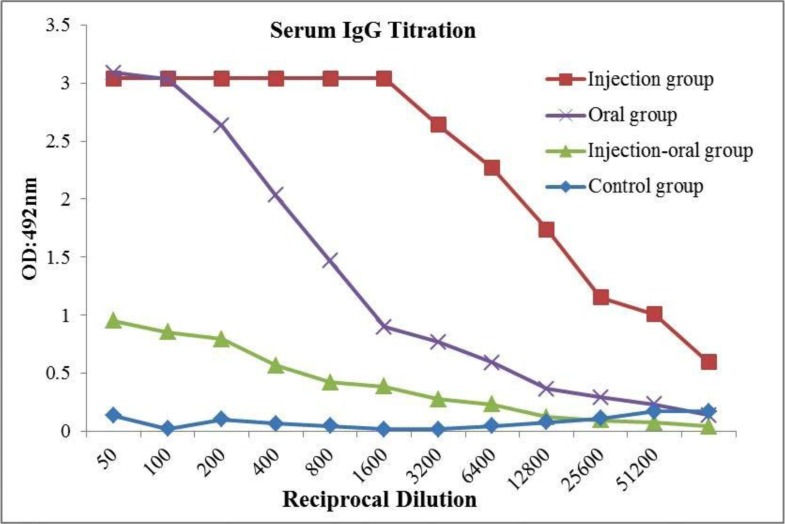
Titration of IgG serum in all mice groups. Experimental mice were divided into four groups based on route delivery, oral-injection, injection, oral and control groups.

**Fig. 7.**
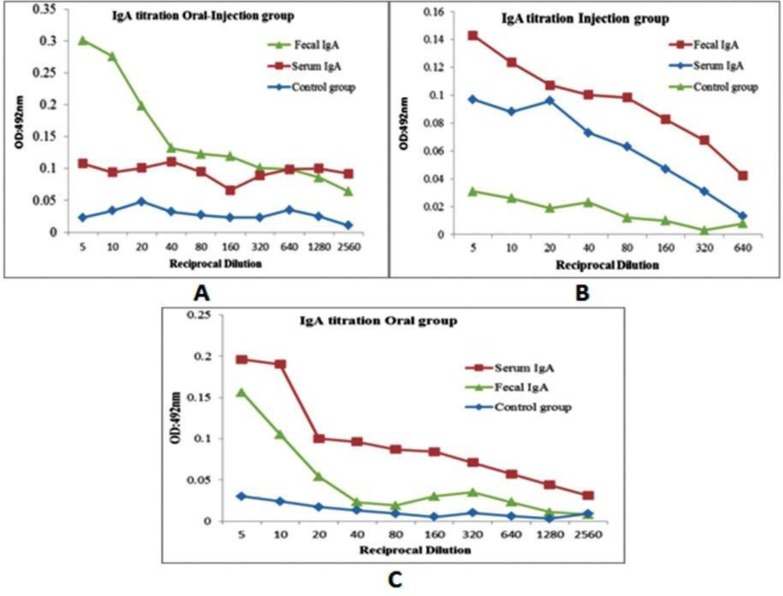
Serum and fecal IgA titration in in all mice groups. Mice were divided to four groups oral-injection, injection, oral and control groups.

Enzyme-linked immunosorbent assay was used to determine the quantity of the specific IgG and IgA antibodies against rStx2B proteins. Figs. 6 and 7 show the specific IgG and IgA antibodies to Stx2B, respectively. This titration was detected as early as the first immunization in the sera from immunized mice which increased significantly after the second booster. There was significant difference (*P*< 0.05) in antibody titers between rStx2B in the first injection.

### Challenge assay.

**Fig. 8.**
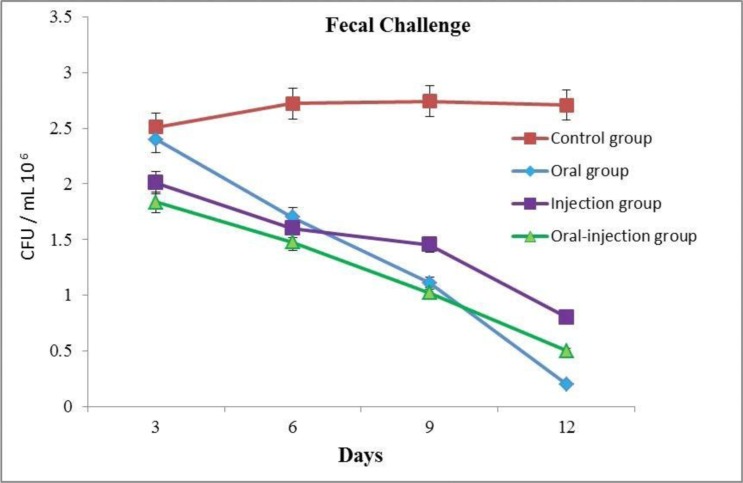
Challenge of immunized mice against *E. coli* O157:H7. Tests and control mice were orally fed 10^10^ *E. coli* O157:H7 and shedding was monitored. The limit of detection for plating was 100 CFU/0.1 g feces.

Results indicated that in the oral group, the highest level of protection was observed in immunized mice (Fig. 8).

## DISCUSSION

Despite the global success of reducing the causes of diarrhea death in the last 30 years, diarrhea remains the second leading cause of death in children under the age of 5 in the world (14, 15). After Shigella, *Escherichia coli* was the highest percentage of pathogens isolated from diarrhea among patients in Iran (16). Enterohemorrhagic *E. coli*, O157: H7 is the most prominent type of *E. coli* which causes diarrhea. The importance of EHEC lies on its pathogenicity. In humans, a wide range of infection can develop from diarrhea without hemorrhage to severe inflammation of the intestines (hemorrhagic colitis of HC). Also, in some cases, it causes urinary hemolytic uremic syndrome (HUS) and even leads to a Thrombotic Thrombocytopenic Purpura (TTP) neurological disorder and patient's death. However, asymptomatic transmission is reported mainly in adults. It seems that after bacterial attachment to the intestinal epithelium, which leads to severe destruction damage (A/E) in epithelial cells of the host, Shiga-toxin is produced and enters the bloodstream and attaches to specific tissue especially the kidneys (17, 18). Infection dose of O157: H7 has been reported at about 1–100 cells. The low dose of infection it possible to be easily transmitted through food or water. A high mortality rate has been reported with EHEC that is different from infections caused by EPEC, ETEC, and EAEC.

Currently, vaccination is one of the most important methods applied to control the O157: H7 in ruminant animals. So far, several vaccines have been designed against the EHEC O157: H7 and experimentally tested. Mostly, traditional vaccines have used surface antigen in cell membrane to attach the host cells. It seems that immunization against one antigen did not completely prevent bacterial attachment. It should be noted that these bacteria use complicated mechanisms for infection. These mechanisms involved the use of several proteins on cell surface (19, 20). The objective of the present study was to prepare a nanoparticle containing Stx2B recombinant protein (a non-toxic immunogenic region) as a vaccine candidate, and then, deliver the nanoparticle in a mouse model orally.

Immunoblotting results indicated that a band of 13 kDa for Stx2b protein and its molecular weight is approximately the same with the protein expressed in the prokaryotic system. This suggests that the protein synthesis machine in the bacterium translated the protein from mature mRNA and there is no deletion in the desired protein. It should be noted that chitosan has disadvantages such as high molecular weight, high viscosity, and low solubility in acid-free solutions. These disadvantages can be addressed via enzymatic hydrolysis and different chemical methods (21). If producing recombinant proteins in *E. coli*, it is necessary to remove bacterial LPS from the final product. This is important when the experimental design is based on immunological assays or evaluating endotoxins on cell lines. LPS is hydrophobic and slightly acidic in neutral pHs. These properties can be utilized to remove endotoxin from protein. We dissolved the inclusion bodies in 8 M urea buffer and passed the protein from a column which bound the protein to an ion exchanger. At pH=8.0 on a Q-based matrix, most proteins will elute once the salt concentration has reached 300 mM NaCl. Endotoxin on the other hand does not come off the matrix until about 0.5 M NaCl (22). We worked based on this protocol and after that we dialyzed and examined for the presence of LPS with LAL test.

Stx2 is the most pathogenic toxin and antibody against it would protect this toxin which causes HUS. The B subunit represents the binding unit of the toxin and is nontoxic for mammalian cells and can be used for cancer therapy (23, 24). Oloomi et al. showed that the B subunit of the shiga toxin could be a good candidate with two peptides as adjuvants for vaccination strategy. These subunit proteins by components such as immunomodulators enhance immunity (25). In this study, we used nanocapsulated Stx2B for improving antigenicity and mucosal immune responses to oral immunization. The nanoparticle size and loading efficiency were estimated at 4.290 nm and 86%, respectively. Kai Zhu et al. used PLA nanoparticles loaded with chitosan as a delivery system for protecting chickens against the Newcastle disease virus (NDV). In this study, the nanoparticle size was estimated to be 699 ± 5.21 nm and the loading efficiency wasd 98% (26). Furthermore, Dilip Pawar et al. and Doavi et al. found that microparticles containing hepatitis B antigen loaded with chitosan and TMC could be considered as a delivery system via the nasal route. In this study, the mean size of nanoparticles was 450 nm (27, 28). Nazarian and colleagues reported that the chimeric protein which was encapsulated in PLGA has nanoparticle size of 252.7 ± 23 and the loading efficiency was 91.96 ± 4.4 (29, 30). Also, according to their study, the best nano-particle size was obtained at concentration 1 mg/ml of chitosan which is in line with our report. Other effective factors in immunization is administration dosage which already reported at 10, 50 and 200 micrograms (31) which was compared with the dosage used in the current study (75 μg) and has good results in immunization of mice. We used the gavage method in oral administration of the antigen which resulted in reduction of degradation of the loaded antigen by mucosal lysozyme. In this regard, the role of chitosan in enhancing antigenic immunization is significant because chitosan protects antigen against proteolytic enzymes.
